# Phylogenetic Analysis of the Endoribonuclease Dicer Family

**DOI:** 10.1371/journal.pone.0095350

**Published:** 2014-04-18

**Authors:** Zeqian Gao, Miao Wang, David Blair, Yadong Zheng, Yongxi Dou

**Affiliations:** 1 State Key Laboratory of Veterinary Etiological Biology, Key Laboratory of Veterinary Parasitology of Gansu Province, Lanzhou Veterinary Research Institute, CAAS, Lanzhou, Gansu, China; 2 School of Marine and Tropical Biology, James Cook University, Townsville, Qld, Australia; Beckman Research Institute of the City of Hope, United States of America

## Abstract

Dicers are proteins of the ribonuclease III family with the ability to process dsRNA, involved in regulation of gene expression at the post-transcriptional level. Dicers are conserved from basal metazoans to higher metazoans and contain a number of functional domains that interact with dsRNA. The completed genome sequences of over 34 invertebrate species allowed us to systematically investigate Dicer genes over a diverse range of phyla. The majority of invertebrate Dicers clearly fell into the Dicer1 or Dicer2 subfamilies. Most nematodes possessed only one Dicer gene, a member of the Dicer1 subfamily, whereas two Dicer genes (Dicer1 and Dicer2) were present in all platyhelminths surveyed. Analysis of the key domains showed that a 5′ pocket was conserved across members of the Dicer1 subfamily, with the exception of the nematode *Bursaphelenchus xylophilus*. Interestingly, *Nematostella vectensis* DicerB grouped into Dicer2 subfamily harbored a 5′ pocket, which is commonly present in Dicer1. Similarly, the 3′ pocket was also found to be conserved in all Dicer proteins with the exceptions of *Schmidtea mediterranea* Dicer2 and *Trichoplax adherens* Dicer A. The loss of catalytic residues in the RNase III domain was noted in platyhelminths and cnidarians, and the ‘ball’ and ‘socket’ junction between two RNase III domains in platyhelminth Dicers was different from the canonical junction, suggesting the possibility of different conformations. The present data suggest that Dicers might have duplicated and diversified independently, and have evolved for various functions in invertebrates.

## Introduction

Small regulatory RNA pathways are highly conserved mechanisms present in most eukaryotic organisms and play an important role in post-transcriptional gene regulation. The gene-regulatory function of microRNAs (miRNAs) and short interfering RNAs (siRNAs) is mainly through translational repression or degradation of cytoplasmic mRNAs by an RNA-induced silencing complex (RISC). miRNA and siRNA pathways share a common RNase III processing enzyme, Dicer, and together with other proteins it constitutes RISC for gene transcriptional repression [1]. Dicer is responsible for recognizing a hairpin (in pre-miRNA) or long double-strand RNA (dsRNA), and processing them into miRNA-miRNA* duplexes or siRNA duplexes [2]. These small RNA duplexes are converted to a single-stranded form and bound to Argonaute (AGO), a key component of RISC, through a process coordinated by Dicer and other RNA-binding proteins [3]. Then small RNAs target specific mRNA sequences, leading to cleavage or translational repression of these [4].

Dicer proteins are present in many eukaryotic organisms including plants, fungi, and metazoans [5], [6]. Vertebrates and nematodes have only one Dicer gene (Dicer1), whereas insects and flatworms possess two, (Dicer1 and Dicer2). Dicers normally contain a number of functional domains: an N-terminal DEAD box, an RNA helicase domain, a Dicer dimer domain, a Piwi-Argonaute-Zwille (PAZ) domain, two RNase III domains and a dsRNA binding domain [7], [8]. The crystal structure of Dicer from *Giardia intestinalis* revealed that the PAZ domain was responsible for binding of the 3′ terminus of dsRNA [9]. After the 3′ end bound to the PAZ domain, pre-miRNAs or dsRNAs are cleaved by the two RNase III domains which form a single dsRNA processing center through intramolecular dimerization [10]. In Dicer1, binding of the PAZ domain to the 3′ terminus of pre-miRNA is crucial for orienting the RNase III domains for cleavage, however, recent publications have revealed that 5′ terminus recognition of pre-miRNAs is also important for mature miRNAs synthesis [11], [12].

Previous studies have focused on Dicers of plants and model organisms, little is known about Dicers of invertebrates. The recent availability of genome sequences of over 34 invertebrate species from 10 phyla, including 1 choanoflagellate, 2 cnidarians, 1 placozoan, 2 annelids, 1 mollusc, 7 platyhelminths, 7 nematodes, 10 arthropods, 1 echinoderm and 3 chordates, have allowed us to perform an extensive phylogenetic analysis of Dicers.

## Materials and Methods

### Acquisition of sequence

For some well-annotated genomes, Dicer sequences were directly retrieved from the databases. In addition, BLASTP and TBLASTN were performed to search against their databases using *Drosophila melanogaster* Dicers (NP_524453 and NP_523778), *Caenorhabditis elegans* Dicer (NP_498761) or *Schistosoma mansoni* Dicers (Smp_169750.1 and Smp_033600) as query sequences. An E-value of 1×e-10 was used as a cutoff in BLAST searches and the hits were filtered to keep only those with at least 25% identity to the query sequence. Protein functional domains were identified using Pfam database and SMART database [13], [14]. The species names, abbreviations and accession numbers are provided in Table 1.

**Table 1:** Distribution of endoribonuclease Dicer genes in invertebrates.

Species	Species Abbreviation	Gene Symbol	Accession Number	Length^a^	Data origin^b^
**Choanoflagellatea**					
* Monosiga brevicollis*	Mbrev	/	/	/	/
**Cnidaria**					
* Nematostella vectensis*	NvecA	Sca_186	ABZ10549^c^	1099	NCBI JGI
	NvecB	167046039	ABZ10551^c^	1469	
* Hydra magnipapillata*	HmagA	HYDRA_212274	Hma2.212274	835	Metazome
	HmagB	HYDRA_205202	Hma2.205202	769	
	HmagC	HYDRA_222700	Hma2.222700	565	
**Placozoa**					
* Trichoplax adhaerens*	TadhA	TRIADDRAFT_18215	XP_002108754	1018	NCBI JGI
	TadhB	TRIADDRAFT_51673	XP_002107798	1418	
	TadhC	TRIADDRAFT_51985	XP_002107959	685	
	TadhD	TRIADDRAFT_52058	XP_002108012	915	
	TadhE	DCL-E	ABZ10547^c^	707	
**Annelida**					
* Capitella teleta*	Ctel	CAPTEDRAFT_223153	ELU12939	1651	NCBI
* Helobdella robusta*	Hrob	estExt_Genewise1.C_710069	103772	1316	JGI
**Mollusca**					
* Lottia gigantea*	Lgig	gw1.12.81.1	61365	1800	JGI
**Platyhelminthes**					
* Schmidtea mediterranea*	Smed1	Dicer-1	ASA.00018.01	1328	SmedGD
	Smed2	Dicer-2	mk4.000125.07.01	1589	
* Schistosoma mansoni*	Sman1	Dicer-1	Smp_169750.1	2319	Sanger
	Sman2	Dicer-2	Smp_033600	928	
* Schistosoma japonicum*	Sjap1	Dicer-1	Sjp_0069770	2480	Sanger
	Sjap2	Dicer-2	Sjp_0043700	923	
* Echinococcus granulosus*	Egra1	Dicer-1	EgrG_000085200	1904	Sanger
	Egra2	Dicer-2	EgrG_000181800	1924	
* Echinococcus multilocularis*	Emul1	Dicer-1	EmuJ_000085200	1906	Sanger
	Emul2	RNC3.1	EmuJ_000180900	1812	
	Emul2	RNC3.2	EmuJ_000181800	1929	
* Hymenolepis microstoma*	Hmic1	Dicer-1	HmN_000252400	1897	Sanger
	Hmic2	Endoribonuclease Dicer	HmN_000200100	1183	
* Taenia solium*	Tsol1	Dicer-1	TsM_000872800	1890	Sanger
	Tsol2	Endoribonuclease Dicer	TsM_000756400	829	
**Nematoda**					
* Caenorhabditis elegans*	Cele	Dicer-1	NP_498761	1910	NCBI
* Bursaphelenchus xylophilus*	Bxyl	Dicer-1	BUX_s00116.153	1040	Sanger
* Pristionchus pacificus*	Ppac	Dicer-1	WBGene00096444	2769	WormBase
* Strongyloides ratti*	Srat	Dicer-1	g5271	1824	WormBase
* Brugia malayi*	Bmal	Dicer-1/Bm5026	WBGene00225287	1930	WormBase
* Trichinella spiralis*	TspiA	Tsp_01223	XP_003377020	2029	NCBI
	TspiB	Tsp_00125	XP_003375890	1903	NCBI
* Loa loa filariasis*	Lloa	LOAG_02227	XP_003137813	1928	NCBI
**Arthropoda**					
* Daphnia pulex*	DpulA	DAPPUDRAFT_308316	EFX72380	1979	NCBI JGI
	DpulB	DAPPUDRAFT_329028	EFX69538	1459	
	DpulC	DAPPUDRAFT_309030	EFX86072	1604	
* Pediculus humanus corporis*	Phum	Dcier-1	XP_002429494	2179	NCBI
* Tribolium castaneum*	Tcas1	Dicer-1	XP_968993	1865	NCBI
	Tcas2	Dicer-2	NP_001107840	1623	
* Nasonia vitripennis*	Nvit1	Dicer-1	XP_001605287	1917	NCBI
	Nvit2	Dicer-2	XP_001602524	1450	
* Acyrthosiphon pisum*	ApisA	LOC100159500	XP_001943370	1626	NCBI
	ApisB	LOC100166428	XP_001945890	1691	
* Drosophila melanogaster*	Dmel1	Dicer-1	NP_524453	2249	NCBI FlyBase
	Dmel2	Dicer-2	NP_523778	1772	
* Anopheles gambiae*	Agam1	AgaP_AGAP002836	XP_003436256	2336	NCBI
	Agam2	AgaP_AGAP012289	XP_320248	1672	
* Aedes aegypti*	Aaeg1	AaeL_AAEL006794	XP_001652212	1658	NCBI
	Aaeg2	AaeL_AAEL001612	XP_001659747	2193	
* Culex pipiens quinquefasciatus*	Cpip1	CpipJ_CPIJ003169	XP_001844757	2270	NCBI
	Cpip2	CpipJ_CPIJ010534	XP_001855187	1165	
**Echinodermata**					
* Strongylocentrotus purpuratus*	Spur	LOC586001	XP_790894	1850	Metazome
**Chordata**					
* Branchiostoma floridae*	Bflo	BRAFLDRAFT_202604	XP_002610617	1868	NCBI
* Ciona intestinalis*	Ciona	Endoribonuclease Dicer	ENSCINP00000017117	1872	Ensemble
* Saccoglossus kowalevskii*	Skow	Sakowv30031161m.g	Sakowv30031161m	1905	Metazome

a Amino acid length;

b JGI: Joint Genome Institute; NCBI: National Center for Biotechnology Information; SmedGD: *Schmidatea mediterranea* Genome Database; Sanger: Wellcome Trust Sanger Insitute; FlyBase: Drosophila database; SilkDB: silkworm database; Ensemble: Ensemble Genome Browser.

### Sequence alignment and phylogenetic analysis

The data sets contained a total of 58 sequences from 34 species (in a size from 565aa to 2769aa, Text S1). The amino acid sequences of Dicer were aligned by MUSCLE [15] with default parameters and manually optimized by Jalview 2.8 [16]. The alignments were subsequently processed using Gblocks v0.91b [17] for phylogenetic reconstruction, allowing gaps in 1/2 of the sequences. ProtTest 3.2 was applied to find an appropriate model of amino acid substitution for tree building analysis [18]. A maximum likelihood tree was constructed using PhyML 3.0 program [19]. Clade support was calculated using SH-like approximate likelihood ratio test, Bayes likelihood test and bootstrap proportions (500 replicates).

## Results

### Identification and distribution of Dicer genes across invertebrates

The final data sets contained 58 Dicer gene sequences from two cnidarians, one placozoa, two annelids, one mollusc, seven platyhelminths, seven nematodes, eleven arthropods, one echinoderm and three chordates (Table 1). No Dicer homologues were identified in the choanoflagellate *Monosiga brevicollis*. Our results of genomic database searches revealed that one placozoan, two annelids, one mollusc, one echinoderm and three of the chordates investigated possessed only one Dicer1 gene. Each of nematodes had only one Dicer1 gene, except *Trichinella spiralis*, which expressed both Dicer1 and Dicer2 genes. Platyhelminths and arthropods possessed two Dicer genes in their genomes, with the exceptions of *Daphnia pulex* (three genes), *Pediculus humanus corporis* (one gene) and *Echinococcus multilocularis* (three genes).

### Phylogenetic analysis of Dicers

**Figure 1:**
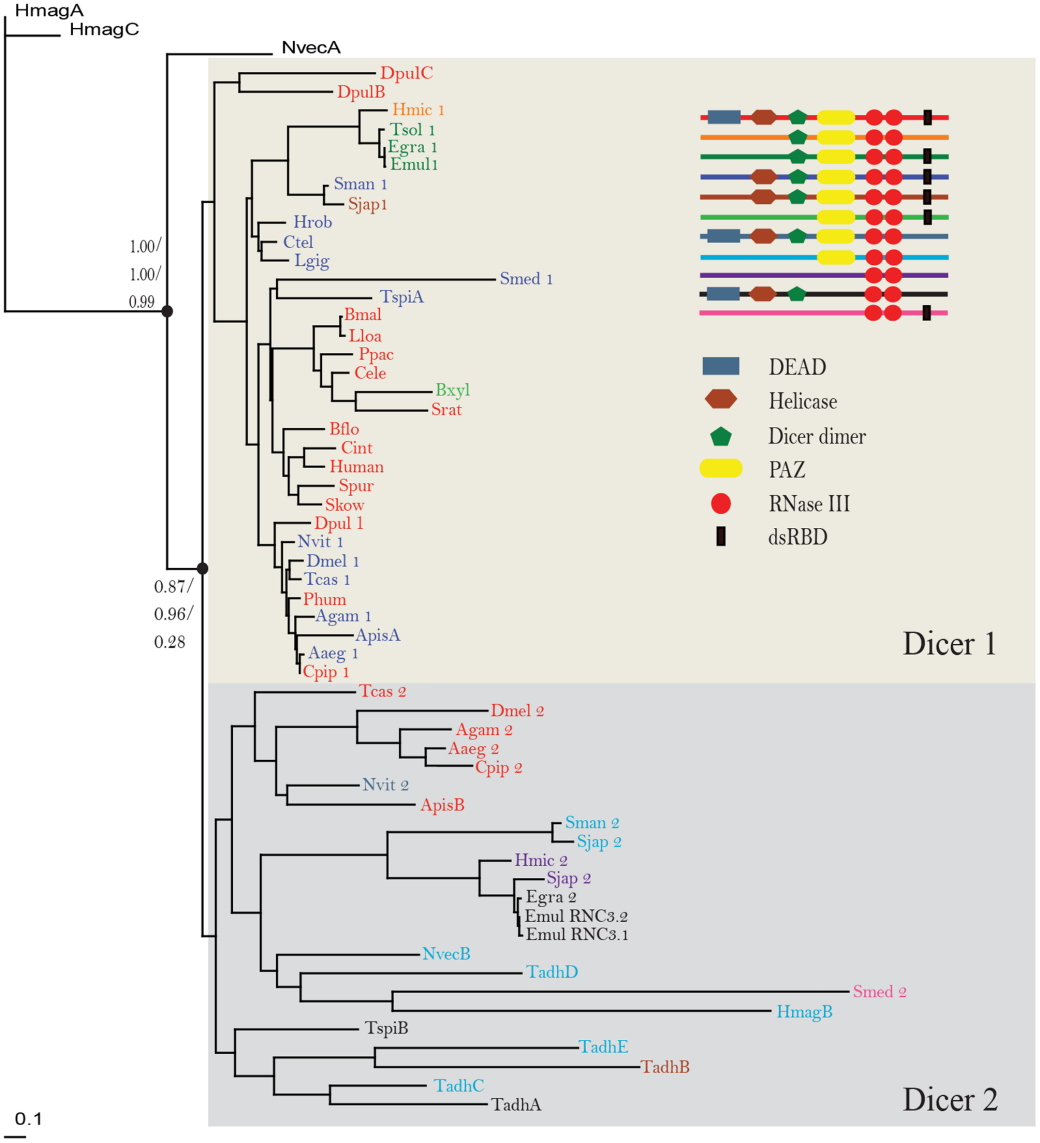
A maximum likelihood tree of invertebrate Dicers. The tree was constructed using maximum likelihood method. Two number sets, 1.00/1.00/0.99 and 0.87/0.96/0.28, at the nodes were SH-like approximate likelihood ratio, Bayes likelihood and bootstrap values, respectively.

As shown in the Maximum likelihood tree (Fig. 1), Dicers of invertebrates were grouped into two lineages: Dicer1 subfamily and Dicer2 subfamily. Almost all of the arthopods and platyhelminths surveyed possessed one member of each of these subfamilies, and annelids, molluscs, nematodes, echinoderms and chordates investigated had only one Dicer gene that belongs to Dicer1 subfamily. The placozoan *Trichoplax adhaerens* had the most copies of Dicer genes in our investigated species; however, all of the five Dicer genes were classed into the Dicer2 subfamily. The two cnidarians *N. vectensis* and *Hydra magnipapillata* each had only one Dicer2 gene, but possessed other Dicer genes that fell outside the two subfamilies.

### Organization of functional domains of Dicer family

We identified the functional domains using the Pfam database and confirmed each inferred domain using the SMART database. As shown in Fig. 1, Dicers had significant variability in domain organization. For instance, Dicers initially characterized in humans are multidomain proteins, consisting of an N-terminal DEAD box, an RNA helicase domain, a Dicer dimer domain, a PAZ domain, two RNase III domains and a dsRNA binding domain [10]. However, *Taenia solium* Dicer2 processed only one RNase III domain. We also observed the loss of the DEAD domain, which contains two RecA-like domains as a catalytic core and can regulate various processes involving RNA [20], in Dicer1 of mollusks, annelids, platyhelminths and most arthropods.

**Figure 2:**
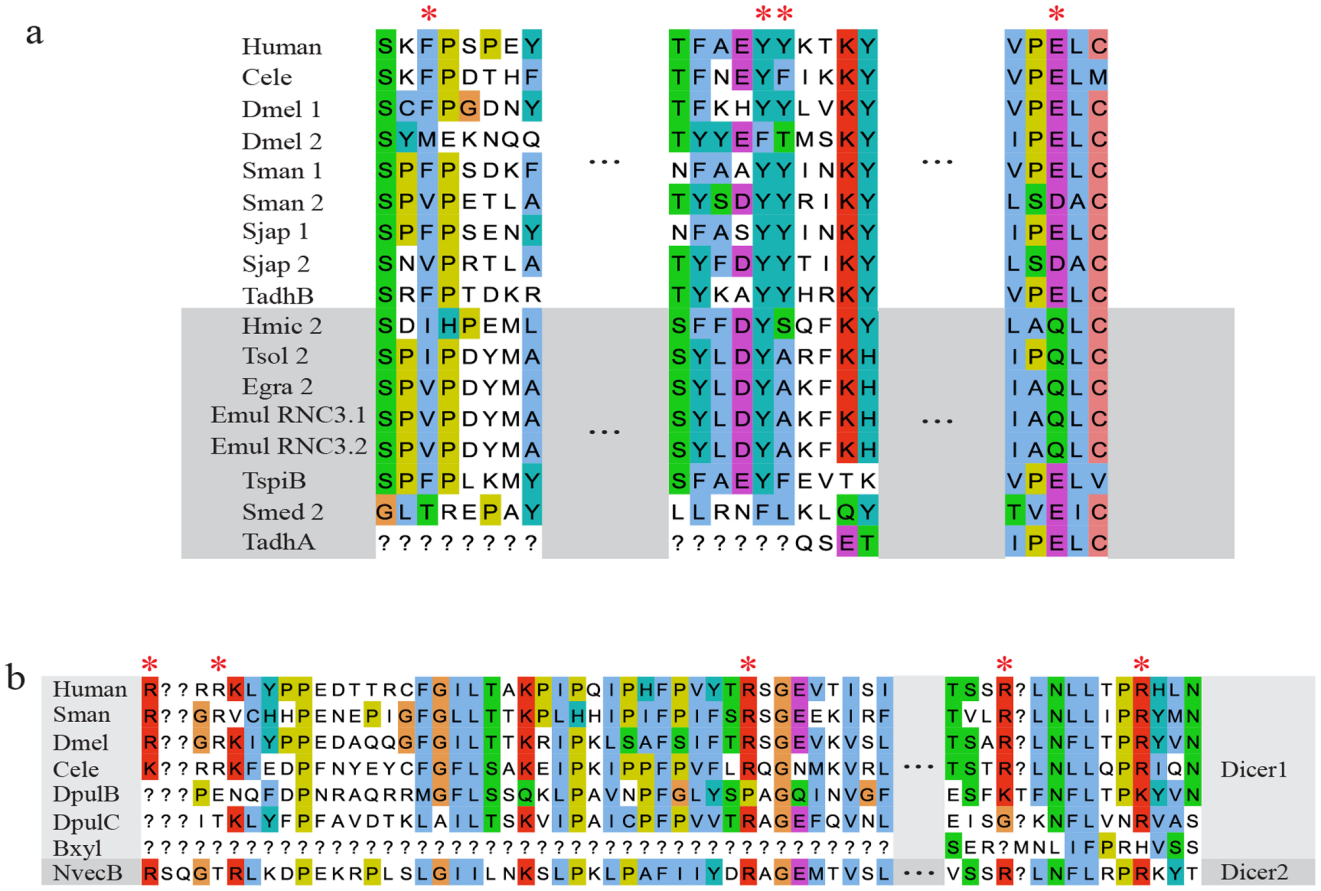
The key residues critical for recognition of 3′ and 5′ pockets. The key residues involved in 3′ pocket (a) and 5′ pocket recognition (b) were indicated using asterisks in red. The Dicers in which a PAZ domain was not identified using Pfam and SMART are highlighted in grey (a). Gaps are filled using question marks (?).

A PAZ domain is an RNA-binding module found in PPD proteins (PAZ and Piwi domain proteins) and Dicer orthologs, and anchors the 2-nucleotide 3′ overhang of dsRNA with its highly conserved binding pocket [10]. After searching annotated domains using Pfam and SMART databases, we did not find the PAZ domain in Dicer2 of the platyhelminths *Schmidtea mediterranea, Hymenolepis microstoma*, *T. solium*, *Echinococcus granulosus*, *E. multilocularis*, the placozoan *T. adhaerens* and the nematode *T. spiralis*. There are two possibilities: the sequences are too divergent to be clearly recognized or they may have lost the PAZ domain during evolution. We therefore aligned the key amino acid residues in the PAZ domain of Dicers in the above species. As shown in Fig. 2a, most of the key residues in the PAZ domain were conserved in Dicer2 sequences with the exceptions of *S. mediterranea* and *T. adhaerens*, indicating the absence of the PAZ domain in those two species. Recently studies have revealed that human Dicer anchors not only the 3′ end but also the 5′ end, and the 5′ end recognition by Dicer is important for the precise and effective biogenesis of miRNAs [12]. A previous study revealed that the 5′ binding residues (Arg778, Arg780 and Arg811 within the N-terminal extension of PAZ domain, and Arg996 and Arg1003 within PAZ domain) were conserved across invertebrate Dicer1 and absent in other Dicers [12]. However, we found that these five key residues were present in *N. vectensis* DicerB, which was classed into the Dicer2 family (Fig. 2b).

**Figure 3:**
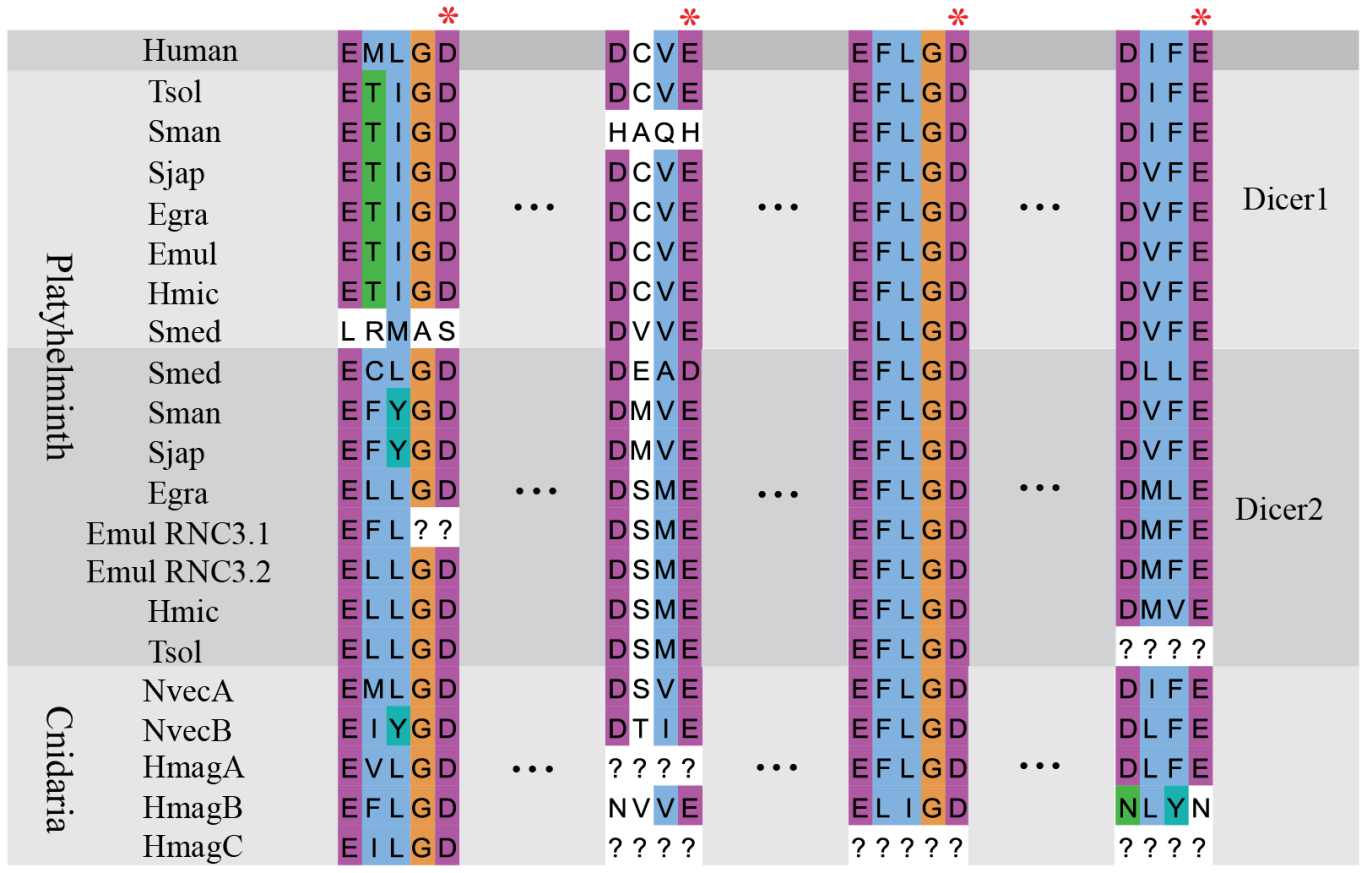
The key residues of RNase III domains. Catalytic residues are marked by red asterisks and gaps filled using question marks (?).

**Figure 4:**
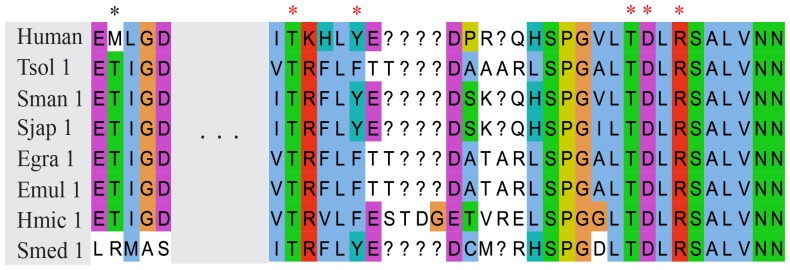
The residues of “ball and socket” junction. The ‘Ball’ residue within RNaseIII a domain is marked by a black asterisk, and the ‘socket’ residues within RNase III b domain are marked by red asterisks. Gaps are filled using question marks (?).

After dsRNA binding to the 5′ and 3′ pockets, two RNase III domains of Dicer cleave targeted molecules. Based on the alignment of Dicer RNase III domain, we found that the catalytic core in most invertebrates was highly conserved (Fig. 3). However, *Schistosoma mansoni* Dicer1, *S. mediterranea Dicer1*, *T. solium* Dicer2 and *E. multilocularis* Dicer RNC3.1 showed variations in this key region (Fig. 3). Compared to platyhelminths, the RNase III domain seemed to be divergent in cnidarians, and most of the key residues were altered in *H. magnipapillata* DicerC (Fig. 3), indicating the possibility of loss of dsRNA cleavage ability. During the cleavage of targeted molecules by sRNAs, two RNase III domains of Dicer form a tight dimer of which the subunit interface is hydrophobic [9]. The crystal structure showed that a tight dimer was formed by two *Aquifex aeolicus* RNase III proteins, each of which possessed only one RNase III domain. A total of 128 hydrophobic interactions (<4.0 Å) were found between the two molecules, whereas only 20 hydrogen bonds/salt bridges existed at the dimer interface. In the dimer, two identical “ball-and-socket” junctions were formed at each end of the interface. The ‘ball’ was the hydrophobic side chain of Phe41 and the ‘socket’ was a cavity formed by side chains of Val52, Val56, Leu67, Ser68, and Lys71 [9]. Subsequent studies showed that a Met1317 within the human RNase III a domain was located in the position of the ‘ball’ residue and the corresponding socket residues Thr1717, Tyr1721, Leu1732, Thr1732 and Arg1736 were located in RNaseIII b domain [21]. Interestingly, we found that the ‘ball’ residue in the RNase III a domain of platyhelminths Dicer1 was replaced by a hydrophilic amino acid-threonine, whereas the corresponding ‘socket’ residues were still conserved (Fig. 4).

## Discussion

In our study, the number of the Dicer genomic loci was variable, from one in several invertebrates to five in *T. adhaerens*. Dicers of invertebrates were clearly classed into two subfamilies, Dicer1 and Dicer2, except for several Dicers from cnidarians. Our results support the model of Dicer evolution in which a eukaryote Dicer may have duplicated independently. Interestingly, Dicer2 of *E. multilocularis* may have undergone duplication after species formation. Mature miRNAs have been identified in all the invertebrates investigated in our research with the exceptions of the choanoflagellate *M. brevicollis* and the placozoan *T. adhaerens* [22]. Similarly, we failed to find Dicer genes and other RISC proteins genes in the genome of *M. brevicollis*, a close known relative of metazoans. However, *T. adhaerens*, a simple known metazoan, possessed five Dicer proteins and all of them belonged to Dicer2 subfamily. These Dicers may constitute an immune defense mechanism against viral infection as placozoans are exposed to a high viral load [23].

Both cnidarians possessed multiple Dicers, and only *N. vectensis* DicerB and *H. magnipapillata* DicerB were classed into Dicer2 group, while the others fell outside the two recognized subfamilies. Recent analysis has revealed that cnidarians express species-specific miRNAs and share few miRNA families with bilateria [24], [25]. These distinct cnidarian Dicers may provide some clues to understanding of the biogenesis of species-specific miRNAs.

The recognition and cleavage of dsRNA by Dicer is a core step in miRNA and siRNA pathways. The 3′ pocket of Dicer is involved in 3′ end binding of dsRNA. The absence of key sites in the pocket in *S. mediterranea* Dicer2 and *T. adhaerens* DicerA could lead to loss of the binding ability. But these two Dicer2 genes may function with the help of other RNA binding protein, such as Drosha [26]. The 5′ pocket is positioned in close proximity to the 3′ pocket on the same surface of Dicer1, and the binding residues of the 5′ pocket are conserved [12]. Interestingly, we found most of the key binding residues of the 5′ pocket, which were previously found only in Dicer1, in *N. vectensis* DicerB that belonged to Dicer2 lineage. It suggests that *N. vectensis* DicerB may retain the bioactivities of Dicer1 as well.

After dsRNA recognition by the conserved pockets, the cleavage of dsRNA is conducted by two RNase III domains [27]. The loss of the catalytic residue-aspartate in *E. multilocularis* Dicer RNC3.1 could reduce its catalytic activity. However, Dicer RNC3.2, a paralogue of Dicer RNC3.1, possessed all the key residues, and therefore it may compensate for the reduced activity of Dicer RNC3.1. The dimerization of RNase III domains creates a catalytic valley which can accommodate a dsRNA substrate. The two “ball and socket” junctions may be responsible in part for the accurate positioning of the catalytic residues in the valley. A Previous study suggested that the ‘ball’ consisted of the hydrophobic side chain of amino acids, and the interaction between ‘ball’ and ‘socket’ was precluded by charged side chain or the absence of any side chain in the position of the ‘ball’ [9]. Interestingly, we found that the ‘ball’ within the RNase III a domain. In platyhelminths Dicer1 proteins contained a hydrophilic side chain of threonine, but the residues of the hydrophobic ‘socket’ were conserved across different species. This substitution may results in reduced affinity between the ‘ball’ and the hydrophobic ‘socket’ and altered conformation.

## Supporting Information

Text S1 **Amino acid sequences of Dicers.**
(TXT)
